# Early harmonies, enduring echoes—how early life experiences and personality traits shape music performance anxiety

**DOI:** 10.3389/fpsyg.2024.1360011

**Published:** 2025-01-22

**Authors:** Ludivine Aubry, Mats B. Küssner

**Affiliations:** ^1^Department of Psychology, Humboldt-Universität zu Berlin, Berlin, Germany; ^2^Department of Clinical Psychology and Psychotherapy, University of Bern, Bern, Switzerland; ^3^Department of Musicology and Media Studies, Humboldt-Universität zu Berlin, Berlin, Germany

**Keywords:** music performance anxiety, musicians’ mental health, parental behavior, attachment style, music teacher’s role, Five-Factor Model, perfectionism, sensory processing sensitivity

## Abstract

Music performance anxiety (MPA) is a deeply personal and often debilitating experience, causing talented musicians to dread the very stages upon which they showcase their art. An increasing number of studies have addressed this anxiety phenomenon, however, definitions vary and the underlying causes remain unclear. According to the DSM-5, MPA is categorized as a specific subtype of social anxiety disorder, with a shared understanding that its development is shaped by predisposing vulnerabilities as well as external stressors and circumstances. This mini-review provides an overview of relevant literature on the multi-dimensional causes of MPA, with a particular focus on early life experiences and personality traits. It aims to address three key challenges in the field by emphasizing the importance of an enhanced investigation of formative life events, recognizing the (potentially) mediating effects of personalities, and highlighting the necessity to explore protective factors. Investigating early life experiences and personality traits in the context of MPA can deepen our understanding of its origin and development, offering valuable perspectives to tailor interventions, prevent the escalation of anxiety, and foster supportive environments conducive to the well-being and professional growth of musicians.

## 1 Introduction

What roles do early life experiences and personality traits play in the development of music performance anxiety (MPA), a multi-faceted and widespread phenomenon commonly experienced by musicians of all skill levels, from hobbyists to professionals (Spahn et al., 2011)? Kenny’s conceptualization of MPA builds on Barlow’s theoretical framework, which posits that three types of vulnerabilities contribute to anxiety development: a generalized biological (hereditary) vulnerability, a generalized psychological vulnerability, and a specific psychological vulnerability (Barlow, 2000, 2002; Kenny, 2011). According to this model, a musician’s inherent traits and formative experiences shape their susceptibility to MPA. A growing body of literature (Fernholz et al., 2019; Kirsner et al., 2023; Papageorgi, 2022) underscores that a comprehensive understanding of MPA requires closer attention to these formative years and traits, while acknowledging their complex interactions. Examining personality traits and early life experiences within this framework helps identify individual vulnerabilities and potential maladaptive coping mechanisms, which lay the groundwork for preventive measures and effective anxiety management interventions. This review aims to provide a structured and critical overview of recent research, highlighting three key areas that demand attention for advancements in the field: the imperative for enhanced exploration of formative life events, understanding the (potentially) mediating effects of personalities, and the necessity to recognize protective factors.

Several researchers have already explored variables contributing to MPA, spanning from genetic factors to performing circumstances and experiences. In particular gender (Hildebrandt et al., 2012; Kenny et al., 2004, 2014; Khalsa et al., 2009; Papageorgi, 2022; Tardif et al., 2023), age (Dempsey and Comeau, 2019; Fehm and Schmidt, 2006; Fishbein et al., 1988; Kenny et al., 2014; Middlestadt, 1990; Osborne et al., 2005; Papageorgi, 2022; Patston and Osborne, 2016; Steptoe and Fidler, 1987), performance setting (Boucher and Ryan, 2011; Papageorgi, 2022; Ryan and Andrews, 2009), instrument type, musical genre (Nusseck et al., 2015; Perdomo-Guevara, 2014), and performance experience (Fehm and Schmidt, 2006; Kenny, 2011; Papageorgi et al., 2013). Beyond the genetic and conceptual factors mentioned above, the depth of personal experiences and individual traits play a crucial role in shaping the onstage experience of musicians. Individual personality traits and early life experiences have been found to influence an individual’s perception and management of psychological distress, such as anxiety (Kotov et al., 2010; Lähdepuro et al., 2019). Some musicians may therefore be inherently more prone to MPA, while others demonstrate greater resilience. Studies have highlighted the role of early life experiences in shaping personality development (Csathó and Birkás, 2018; Kitamura and Fujihara, 2003) and their interconnection with psychosocial factors such as self-esteem, self-efficacy, and social support (Bandura, 1997; Pérez-Fuentes et al., 2019). For instance, a study conducted by Kitamura et al. (1999) showed that individuals who perceive stronger social support tend to display higher levels of extraversion and lower levels of neuroticism. Other findings indicated that extraversion, agreeableness, conscientiousness, and openness to experience were positively associated with self-esteem, whereas neuroticism was found to be a significant negative predictor (Amirazodi and Amirazodi, 2011). Social support, self-esteem, and self-efficacy have also been found to play pivotal roles in shaping musicians’ beliefs about their ability to cope with anxiety and on how to perform well (Chan, 2011; Dempsey and Comeau, 2019; Papageorgi, 2022; Schneider and Chesky, 2011). How early life experiences and personality traits affect MPA’s emergence will be discussed in the following sections.

## 2 Early life experiences

Studies suggest that children exposed to early adverse experiences are more prone to developing anxiety or other mental illnesses (Bick and Nelson, 2016). Therefore, acknowledging the heightened vulnerability during these stages is essential when addressing MPA. Early life experiences can mold core beliefs, influencing self-esteem, self-efficacy, and the overall perspective on performance (Givertz and Segrin, 2014; Kirsner et al., 2023; McPherson et al., 2012; Zarza-Alzugaray et al., 2018). Furthermore, the occurrence of MPA in very young musicians (Boucher and Ryan, 2011) underscores the need to address coping strategies as early as possible, which, correspondingly, relies on the support provided by their environment (Fehm and Schmidt, 2006). Factors explored in the context of early life experiences and MPA include parental behavior, attachment style, the influence of music teachers, and early exposure to music performance. For an overview, see Supplementary Table 1.

### 2.1 Parental behavior

During childhood, parental behavior can exert substantial influence on children’s musical development. McPherson (2008) presents a model that clarifies how parental goals influence various child outcomes, including musical competence and the development of a musical identity. Parents, for instance, who show interest and value their children’s musical activities through supportive words or actions, strengthen the parent-child relationship, provide encouragement and motivation during difficult times, and enhance feelings of competence (McPherson and Davidson, 2002). Moreover, parents often take the lead in initiating young musicians’ careers and are actively involved in their offspring’s musical advancement (Corrigall and Schellenberg, 2015; Upitis et al., 2016). Ryan et al. (2023) noted that young piano students with musically educated parents exhibited higher levels of MPA, suggesting a potential influence of the parents’ expectations and their critical understanding of musical performance. In the course of researching MPA various parenting styles were examined. Notably, perceiving parents as critical, abusive, over-controlling, or overprotective emerged as potential risk factors for experiencing heightened MPA (Aubry and Küssner, 2023; Dobos et al., 2019; Kirsner et al., 2023; Papageorgi, 2022; Wiedemann et al., 2020). A study by Kirsner et al. (2023) explored MPA in the context of early childhood experiences and dysfunctional cognitive schemas. The authors found that when musicians perceived their primary caregivers as failing to meet fundamental emotional needs, it could lead to the development of dysfunctional cognitive schemas, which were subsequently identified as significant predictors of MPA. The fundamental aspect of these schemas, as defined by Young et al. (2003), was characterized as *Impaired Autonomy and Performance*. Individuals with this schema may struggle with a persistent sense of incompetence, fear of failure, or a heightened sensitivity to perceived shortcomings, impacting self-esteem, decision-making, and overall performance.

### 2.2 Early attachment

Attachment style, which refers to an individual’s patterns of emotional bonding and connection developed in early childhood through interactions with caregivers, has been linked to MPA (Kenny, 2011). These early attachment patterns shape one’s approach to intimacy and relationships throughout life (Bowlby, 1969; Karavasilis et al., 2003). However, there are only a limited number of publications exploring this area in relation to MPA (Kenny and Holmes, 2015; Kenny and Holmes, 2018; Wiedemann et al., 2020). Wiedemann et al.’s (2020) findings suggest that musicians characterized by dismissive or secure attachment styles exhibit lower anxiety levels compared to those with preoccupied or anxious attachment styles. Individuals exhibiting a dismissive or secure attachment style may possess a greater sense of confidence, self-assurance, and emotional security, leading to a reduced susceptibility to performance anxiety. In contrast, individuals displaying a preoccupied or anxious attachment style may experience heightened performance anxiety due to increased self-doubt, concerns about external evaluation, or a lack of confidence in their abilities. A published case report further demonstrated the relationship between insecure attachment and the occurrence of MPA. A young musician’s unresolved early attachment ruptures contributed to vocal difficulties and heightened anxiety during musical performances (Kenny and Holmes, 2015).

### 2.3 The role of music teachers

The importance of the music teacher’s role in the context of MPA has been underscored in various studies. Music teachers serve as influential guides during key stages of musical education. They shape students’ perceptions, self-efficacy, and coping mechanisms, thereby influencing their pupils’ vulnerability to experiencing and ability to manage MPA (MacAfee and Comeau, 2023; Gill et al., 2022). However, it seems that music teachers do not always adequately address MPA during performance preparation. Fehm and Schmidt (2006) identified the significant influence of teachers on anxiety in music students aged 15–19, which was linked to their expertise and the perceived judgments they made. The students further conveyed a desire for more support in managing performance-related anxiety, emphasizing the importance of candid discussions about experienced distress and highlighting the necessity to provide more practical techniques such as relaxation or performance skills. Ryan et al. (2021) found comparable outcomes revealing that although a considerable number of piano students experienced nervousness during lessons, less than half mentioned that their teachers tackled concerns related to performance preparation, memorization strategies or MPA management. Moreover, they noted that students who experience negative emotions after lessons often link it to a sense of having disappointed their teacher. Ryan and Andrews (2009) investigated choral singers from seven choirs, stating that the conductors’ attitude and behavior during rehearsals emerged as a crucial factor influencing the singers’ experiences of performance anxiety.

### 2.4 Early exposure to music performance

Although the literature presents differing views on how performance experience influences MPA (Fehm and Schmidt, 2006; Kenny, 2011; Papageorgi et al., 2013; Sârbescu and Dorgo, 2014), a recent study highlighted that the onset age of musical training exert a favorable impact on MPA. According to Zarza-Alzugaray et al. (2018) musicians who commence their musical training at or before the age of seven typically report lower levels of performance-related anxiety compared to those who initiate training later in life. Cultivating familiarity and comfort with musical elements, instruments, and performance environments may reduce anxiety and facilitates individuals’ navigation through such events. Early skill development might enhance confidence and competence in performance, potentially reducing anxiety arising from the fear of making mistakes or facing negative judgment. However, in addition to the onset age of musical training, the nature of the experience appears to be crucial. Kenny and Holmes (2018) revealed in their qualitative research that humiliating sensitizing experiences during adolescence, such as memory lapses associated with feelings of shame, are linked to heightened MPA. Similarly, Osborne and Kenny (2008) discovered that music students who reported having a negative performance experience scored notably higher on MPA.

## 3 Personality traits

Musicians’ personality traits have been studied with regard to musical preferences (Rentfrow and McDonald, 2009), training and involvement (Corrigall and Schellenberg, 2015), genius (McCrae and Greenberg, 2014), and musical sophistication (Greenberg et al., 2015). Personality is further linked to the experience of flow during musical activities and performances (Antonini Philippe et al., 2022; Biasutti, 2017; Cohen and Bodner, 2019; Forbes, 2021). Whereas individuals displaying higher levels of Openness, Emotional Stability, Extraversion, and Conscientiousness are more inclined to encounter the psychological state of flow (Butkoviæ et al., 2015; Gözmen and Aşçı, 2016; Tan et al., 2021), suffering from MPA decreases the probability of experiencing flow (Cohen and Bodner, 2019, 2021). Flow is thus fundamental for musicians dealing with MPA, as it enhances performance quality and reduces anxiety-related symptoms, contributing to a more fulfilling and enjoyable musical experience (Spahn et al., 2021). While studies on trait anxiety have offered significant insights into MPA (Osborne and Kenny, 2008; Niering et al., 2023; Thomas and Nettelbeck, 2014), the following sections will concentrate on personality traits that reveal more specific influences on this phenomenon. While trait anxiety helps explain general predispositions to anxiety, it does not capture the distinct ways in which individuals experience and respond to performance-related stress. In contrast, the Five-Factor Model, perfectionism, and sensory processing sensitivity (SPS) might directly shape MPA through specific mechanisms. For example, openness to experience may help performers reframe anxiety as a source of motivation and growth; perfectionism may heighten anxiety by enforcing unattainable performance standards; and SPS might amplify stress through an increased sensitivity to environmental stimuli, such as audience feedback or performance settings. By concentrating on these personality dimensions, we can uncover distinct subtypes of MPA, identify critical stress triggers for different individuals, and provide tailored strategies that address the unique interplay of personality and performance-related stress (see overview in Supplementary Table 2).

### 3.1 Five-Factor Model

The Five-Factor Model, commonly referred to as the Big Five personality traits, is a widely accepted framework in psychology, encompassing Openness (the inclination to embrace new ideas and experiences), Conscientiousness (organized and goal-directed behavior), Extraversion (sociability and assertiveness), Agreeableness (cooperativeness and empathy), and Neuroticism (emotional instability and lack of resilience) (Digman, 1990; Goldberg, 1993). A study on the Five-Factor Model and performance anxiety revealed that Neuroticism and Conscientiousness predicted performance anxiety positively, while Extraversion predicted performance anxiety negatively. Openness correlated negatively with performance anxiety but lacked predictive value, and Agreeableness showed no correlation (Özdemir and Dalkıran, 2017). Although research on the Five-Factor Model is limited in the context of MPA, one of its components—Neuroticism—features widely in the literature about MPA. Neuroticism reflects the extent to which an individual experiences negative emotions and emotional instability. It correlates positively with various mental health issues and appears to negatively predict life quality and longevity (Lahey, 2009). It is therefore not surprising that musicians’ with neurotic tendencies seem to be in general more susceptible to MPA (Hodapp et al., 2009; Rae and McCambridge, 2004; Sadler and Miller, 2010; Smith and Rickard, 2004; Spahn et al., 2024; Steptoe and Fidler, 1987). Neurotic traits may intensify emotional responses to MPA stressors, contributing to persistent rumination on potential mistakes, heightening the fear of failure, and increasing concerns about perceived judgment and criticism. This, in turn, further fuels the cycle of anxiety in the intricate realm of music performance.

### 3.2 Perfectionism

Perfectionism is a personality trait characterized by a persistent striving for flawlessness, an elevated tendency for self-criticism, and the establishment of unrealistically high standards (Flett and Hewitt, 2002). In a competitive environment such as the music industry, the fear of making mistakes and the constant pursuit of excellence motivates musicians to strive for perfection. Studies have shown that perfectionism appears to already be common among young musicians (Patston and Osborne, 2016; Stoeber and Eismann, 2007) and findings reveal positive correlations between MPA and perfectionism (Diaz, 2018; Dobos et al., 2019; Kenny et al., 2004; Kobori et al., 2011; Langendörfer et al., 2006; McNeil et al., 2022; Mor et al., 1995; Papageorgi, 2022; Sarıkaya and Kurtaslan, 2018; Sinden, 1999). Patston and Osborne (2016) highlight a more pronounced developmental trajectory for females compared to males and demonstrate that while developmental pathways are similar for both genders in late childhood, they appear to diverge during early adolescent. Moreover, as musicians gain experience, levels of both MPA and perfectionism tend to ascend. Notably, MPA research underscores the need to differentiate between positive and negative aspects of perfectionism. Stoeber and Eismann (2007) demonstrated that responding negatively to imperfection correlated positively with performance anxiety, while the pursuit for perfection aligned with musical effort and accomplishment. These findings were supported by Butkoviæ et al. (2022) who identified a positive correlation solely between maladaptive perfectionism and MPA, without any observed link to adaptive perfectionism. Dobos et al. (2019) found positive associations with only four out of six perfectionism subscales, including Parental Criticism and Doubts about Actions. Patston and Osborne (2016) further emphasized a particularly significant positive relationship between MPA and the subscale Concern over Mistakes, emphasizing the diverse nature of perfectionism.

### 3.3 Sensory processing sensitivity

Sensory processing sensitivity (SPS) is a personality trait characterized by a heightened awareness and responsiveness to sensory stimuli. Individuals experiencing high SPS process information on a much deeper level, experience intensified emotional reactions, and may find themselves more easily overwhelmed by intense sensory input. It is assumed that highly sensitive people are inclined to choose creative professions (Aron, 1997), suggesting that this trait might be especially prevalent among musicians. Highlighting this assumption, Bridges and Schendan (2019) illustrate how individuals with heightened sensitivity tend to demonstrate elevated levels of creativity. This occurrence arises from a combination of diverse traits and biological processes that influence the development of neurotransmitter systems, sensitivity mechanisms (especially reduced inhibition), and brain networks responsible for automatic attention and orientation. While SPS has been studied in connection with various aspects of psychological functioning (Aron et al., 2005; Liss et al., 2005, 2008), its association with MPA has been relatively unexplored. However, a recent study examined the relationship between MPA, parenting style and SPS in a diverse sample of 342 musicians, indicating that SPS could be a potential risk factor for experiencing heightened MPA (Aubry and Küssner, 2023).

## 4 Challenges for future research

To advance our knowledge of the impact of early life experiences and personality traits on MPA, we propose to focus on three key areas: examining formative life events, investigating how personality traits (potentially) mediate MPA, and identifying protective factors. Addressing these areas will enhance our ability to identify risk factors and enable us to develop more effective interventions. These points are explored further in the subsequent sections.

### 4.1 Enhanced investigation of formative life events

Research on the impact of early life experiences on MPA is still limited, and there is a pressing need for a deeper exploration of how formative life events and cultural/environmental influences shape the development of MPA. For instance, the absence of research examining the connection between trauma and MPA is particularly striking, given that trauma’s effects have been explored in connection to various aspects of musical practice, including creative expression, performance, memory, and concentration (Swart, 2014; Swart et al., 2010). Trauma has also been linked to anxiety disorders (Heim and Nemeroff, 2001; Kuo et al., 2011; Lochner et al., 2010; Śpila et al., 2008), but its specific impact on performance anxiety remains unexplored. Understanding this mechanism could lead to more targeted, trauma-sensitive interventions that address the unique challenges musicians face when performing under intense fear. Moreover, examining other formative experiences—such as negative peer interactions, social standing, experiences of loss, social isolation and early physical health issues—will broaden our understanding of MPA’s complexity.

### 4.2 Understanding personality’s (potentially) mediating effect

It is crucial to recognize that the trajectory of personality traits is not uniform, with personality and early life experiences being deeply intertwined (Srivastava et al., 2003). This relationship underscores the individual variability in how musicians experience and cope with MPA, highlighting the need to address the holistic development of musicians when exploring MPA in the context of their careers. While the specific connection between early life experiences and personality in relation to MPA has been limitedly explored, findings in other domains suggest that these effects are relevant. For instance, Liu et al. (2021) found that childhood psychological maltreatment is linked to increased neuroticism and reduced coping behavior, contributing to higher levels of social anxiety. Thus, musicians with a history of childhood distress may be more vulnerable to MPA due to the development of anxiety-prone personality traits. Moreover, traits such as perfectionism and sensory processing sensitivity may act as mediators between early life experiences and MPA. For example, musicians who grew up in hypercritical or unsupportive environments may develop perfectionistic tendencies, characterized by an intense fear of failure and a drive for flawless performance, which heightens their susceptibility to MPA. Similarly, individuals with high SPS, tend to react more intensely to all kinds of environmental stimuli. This heightened responsiveness can increase the likelihood of developing anxiety, as they are more affected by stressors such as performance pressure or audience reactions (vulnerability-stress model, Zubin and Spring, 1977). Further empirical studies are essential to shed light on the mediating roles of perfectionism and SPS in the development of MPA.

### 4.3 The exploration of protective factors

Finally, it seems important to not only address how early life experiences can have adverse effects on MPA but also to emphasize factors that can yield positive effects. Currently there are no studies investigating for instance parenting styles that may mitigate the development of MPA. Nevertheless, research in other areas of psychological functioning has indicated that an authoritative parenting approach serves as a preventive measure against anxiety in children. Authoritative parents are responsive to their children’s needs while maintaining clear expectations (Erozkan, 2012; Manoochehri and Mofidi, 2014; Panetta et al., 2014; Pinquart, 2017; Timpano et al., 2015; Wei and Kendall, 2014; Wolfradt et al., 2003; Yaffe, 2018; Yazdani and Daryei, 2016). It would be interesting to find out if this holds true in the context of MPA. Providing a guided, structured and at the same time supportive environment may encourage independence and self-expression contributing positively to a child’s confidence in its musical pursuit. The same applies to the behavior and the role of music teachers. Indeed, several recent studies have focused on strategies that teachers impart to help their students better cope with performance stressors (MacAfee and Comeau, 2023; Huang and Yu, 2022). However, some teachers stated feeling ill-prepared to support pupils in this regard and expressed a need for additional training to effectively address MPA during music lessons (Moura and Serra, 2021; Sieger, 2017). Further research should explore how primary caregivers and teachers can enhance musicians’ overall enjoyment of performances.

## 5 Conclusion

Considering MPA to be multifaceted, early life experiences and personality appear to influence musicians’ responses to performance stress. However, limited research exists on how specific formative experiences (e.g., trauma, negative peer interactions, cultural influences) contribute to MPA’s development. Similarly, the mediating roles of personality traits like perfectionism or SPS remain underexplored, particularly their interplay with early life experiences in creating vulnerability or fostering resilience to MPA. Addressing these gaps is crucial for identifying how foundational experiences and personality traits predispose individuals to MPA or buffer against it. Such knowledge would clarify risk factors and support the development of targeted, evidence-based interventions, especially for musicians with unique vulnerabilities. By uncovering the nuanced interplay between early life experiences and personality traits, we can refine our understanding of MPA, predict susceptibility more accurately, and tailor interventions that empower musicians to navigate performance anxiety with greater confidence and resilience, ultimately supporting their well-being and artistic expression.
