# Supplementary material for: Early harmonies, enduring echoes—how early life experiences and personality traits shape music performance anxiety
Source: Front Psychol. 2025 Jan 22;15:1360011. doi: 10.3389/fpsyg.2024.1360011 (PMC11794320; doi:10.3389/fpsyg.2024.1360011)
Supplement: Supplementary file 1 [file Table_1.docx]

Supplementary Material

**Supplementary Table 1.** *Studies exploring the role of early life experiences in relation to MPA*

| **Reference** | **Title** | **Participants** | **Variable** | **Outcome** | **Ql / Qn** |
| --- | --- | --- | --- | --- | --- |
| Aubry and Küssner (2023) | Music performance anxiety and its relation to parenting style and sensory processing sensitivity | *N* = 342 musicians  aged between 18 and 65 years (*M* = 30.11, *SD* = 9.86) | parenting styles (abusive, over-control, indifference) | Retrospectively perceived abusive and over-controlling parenting were significantly associated with heightened MPA levels. | quantitative  research |
| Fehm and Schmidt (2006) | Performance anxiety in gifted adolescent musicians | *N* = 74 music students aged between 15 and 19 years (*M* = 17.1, *SD* = 1.2) | coping strategies and the need for support | Students expressed a need for increased support by their teachers in managing performance-related anxiety through candid discussions and practical techniques like relaxation or performance skills. | quantitative  research |
| Kenny and Holmes (2015) | Exploring the attachment narrative of a  professional musician with severe performance anxiety: A case report | *N* = 1 professional musician aged 26 | attachment experience | The musician experienced past and present attachment ruptures which became apparent through symptoms associated with MPA. | qualitative research |
| Kenny and Holmes (2018) | Attachment quality is associated with music performance anxiety in professional musicians: An exploratory narrative study | *N* = 10 professional musicians aged between 27 and 56 (*M* = 43.8, *SD* = 9.43) | musical and relational life experiences | Early attachment trauma appeared to be a relevant factor contributing to the development of MPA. | qualitative research |
| Kirsner et al. (2023) | Music performance anxiety: The role of early parenting experiences and cognitive schemas | *N* = 100 musicians aged between 18 and 65 years or older | early parenting experiences and patterns of dysfunctional cognitive schemas | The neglect of core emotional needs by primary caregivers can result in the development of dysfunctional cognitive schemas (Failure to Achieve, Dependence/Incompetence, and Vulnerability to Harm or Illness), which, in turn, have been identified as substantial predictors of MPA. | quantitative  research |
| Papageorgi (2022) | Prevalence and predictors of music performance anxiety in adolescent learners: Contributions of individual task-related and environmental factors | *N* = 410 adolescent musicians aged between 12 and 19 years (*M* = 15.33) | factors affecting MPA levels such as individual characteristics task-efficacy, or the performance environment | Higher levels of MPA were associated with perceiving parents as critical. | quantitative  research |
| Ryan and Andrews (2009) | An investigation into the choral singer’s experience of music performance anxiety | *N* = 201 members of semiprofessional choirs aged between 17 and 70 years | experience with conductors in regard to MPA | The choral singers' encounter with performance anxiety was notably influenced by the conductor as a primary factor. | quantitative  research |
| Ryan et al. (2021) | Performance preparation, anxiety, and the teacher. Experiences of adolescent pianists | *N* = 62 adolescent piano students aged between 11 and 17 years (*M* = 13.63, *SD* = 2.01) | student-teacher relationship | Only fewer than half of the students reported that teachers addressed issues related to MPA management. Further, students who experienced negative emotions after lessons often associated it with a sense of disappointing their teachers, and the presence of a teacher at a performance was perceived by some students as particularly stressful. | quantitative  research |
| Ryan et al. (2023) | Practice, performance, and anxiety: A pilot study on student perception of parental involvement and formal music lessons | *N* = 62 piano students aged between 11 and 17 years (*M* = 13.63, *SD* = 2.01) | parental involvement | Parents’ prior music education was significantly correlated with children’s MPA, whereas parent involvement in music studies and responses to weak performances did not show a significant association with MPA scores. | quantitative  research |
| Wiedemann et al. (2020) | The role of retrospectively perceived parenting style and adult attachment behaviour in music performance anxiety | *N* = 82 music students aged between 18 and 33 years (*M* = 23.5, *SD*  = 3.4) | parenting style and adult attachment behavior | Parenting style and adult attachment behavior were associated with MPA in unconditional analysis. Dismissive or secure attached participants exhibited lower levels of MPA, while preoccupied or anxious attached participants demonstrated higher MPA levels. | quantitative  research |
| Zarza-Alzugaray et al. (2018) | Music performance anxiety in adolescence and early adulthood: Its relation with the age of onset in musical training | *N* = 646 music students aged between 16 and 51 years (*n_1_* = 437, *M* = 22.64, *SD* = 4.73) and 8 and 46 years (*n_2_* = 209, *M* = 12.09, *SD* = 4.22) | relation between MPA and the age of onset in musical training | Musicians who started their musical training at or before the age of seven frequently reported lower levels of performance-related anxiety than those who commenced training later in life. | quantitative  research |
