# Supplementary material for: Early harmonies, enduring echoes—how early life experiences and personality traits shape music performance anxiety
Source: Front Psychol. 2025 Jan 22;15:1360011. doi: 10.3389/fpsyg.2024.1360011 (PMC11794320; doi:10.3389/fpsyg.2024.1360011)
Supplement: Supplementary file 2 [file Table_2.docx]

Supplementary Material

**Supplementary Table 2.** *Studies exploring the role of personality traits in relation to MPA*

| **Reference** | **Title** | **Participants** | **Personality Trait** | | **Outcome** |
| --- | --- | --- | --- | --- | --- |
| Aubry and Küssner (2023) | Music performance anxiety and its relation to parenting style and sensory processing sensitivity | *N* = 342 musicians  aged between 18 and 65 years (*M* = 30.11, *SD* = 9.86) | sensory processing sensitivity | Sensory processing sensitivity was positively associated with the experienced extent of MPA. | |
| Butković et al. (2022) | Music performance anxiety and perfectionism in Croatian musicians | *N* = 239 musicians aged between 16 and 64 years (*M* = 28.08, *SD* = 12.37) | perfectionism | Maladaptive perfectionism predicted MPA positively. | |
| Diaz, F. M. (2018) | Relationships among meditation, perfectionism, mindfulness, and performance anxiety among collegiate music students | *N* = 255 collegiate-level musicians | perfectionism | Self-oriented and socially prescribed perfectionism were linked to elevated scores in MPA. | |
| Dobos et al. (2019) | Music performance anxiety and its relationship with social phobia and dimensions of perfectionism | *N* = 100 musicians aged between 15 and 35 years (*M* = 23, *SD* = 9.8) | perfectionism | Four out of six perfectionism subscales were substantial positive predictors of MPA (Concern over Mistakes, Doubts about Actions, Personal Standards, Parental Criticism). Parental Expectations and Preference for Organization predicted MPA negatively. | |
| Hodapp et al. (2009) | Arbeitsbedingungen, gesundheitliche Beschwerden und Aufführungsängste bei professionellen Orchestermusikern | *N* = 150 musicians from professional symphony and opera orchestras (*n* = 122) and amateur orchestras (*n* = 28) | neuroticism | Neuroticism was positively associated with MPA. | |
| Kenny et al. (2004) | Music performance anxiety and occupational stress amongst opera chorus artists and their relationship with state and trait anxiety and perfectionism | *N* = 32 chorus artists aged between 28 and 61 years (*M* = 41.39, *SD* = 9,79) | perfectionism | Perfectionism and MPA showed a positive connection. Nevertheless, perfectionism did not contribute to predicting MPA when accounting for the influence of state and trait anxiety, as well as when considering solo or choral musical performance scales. | |
| Kobori et al. 2011) | Traits and cognitions of perfectionism and their relation with coping style, effort, achievement, and performance anxiety in Japanese musicians | *N* = 275 professional and amateur Japanese musicians (*M* = 28.40, *SD* = 10.64) | perfectionism | Achievement showed an association with perfectionism traits, while performance anxiety only exhibited a weak connection and was moderately linked to specific perfectionism cognitions, such as the Pursuit of Perfection and Concern over Mistakes. The latter explained unique variance in performance anxiety. | |
| Langendörfer et al. (2006) | Personality and performance anxiety among professional orchestra musicians | *N* = 122 orchestra musicians aged between 25 and 64 years (*M* = 42.02, *SD* = 10.08) | perfectionism and neuroticism | Socially pre-scribed perfectionism was predictive of performance anxiety during rehearsals, whereas self-oriented perfectionism predicted performance anxiety before a performance. Neuroticism showed significant correlations with performance anxiety subscales, but did not function as a predictive variable. | |
| McNeil et al. (2022) | Investigating the moderating role of coping style on music performance anxiety and perfectionism | *N* = 118 musicians aged between 18 and 76 years (*M* = 42, *SD* = 14.68) | perfectionism | Personal standards perfectionism and evaluative concerns perfectionism were both associated with MPA. | |
| Mor et al. (1995) | Perfectionism, control, and components of performance anxiety in professional artists | *N* = 87 professional performing artists (*n* = 49 classical musicians) | perfectionism | Self-oriented and socially prescribed perfectionism were linked to performance anxiety. The findings emphasized that perfectionism is not only connected to heightened debilitating anxiety but also to a reduced presence of facilitating anxiety. | |
| Özdemir and Dalkiran (2017) | Identification of the predictive power of five factor personality traits for individual instrument performance anxiety | *N* = 256 undergraduate music students | Five Factor Personality Traits (Neuroticism, Conscientiousness, Openness, Agreeableness, Extroversion) | Neuroticism and Conscientiousness predicted performance anxiety positively, whereas Extroversion predicted performance anxiety negatively. Openness and Agreeableness were not found to be significant predictors of MPA. | |
| Patston and Osborne (2016) | The developmental features of music performance anxiety and perfectionism in school age music students | *N* = 526 musicians aged between 9 and 17 years (*M* = 12.56, *SD* = 1.79) | perfectionism | MPA and perfectionism were positively associated, particularly in relation to the subscale Concern over Mistakes. | |
| Rae and McCambridge (2004) | Correlates of performance anxiety in practical music exams | *N* = 120 musicians aged between 15 and 18 years  (*M* = 16.5, *SD* = 0.97) | neuroticism | Elevated neuroticism levels were associated with increased MPA. | |
| Sadler and Miller (2010) | Performance anxiety: A longitudinal study of the roles of personality and experience in musicians | *N* = 37 undergraduate music performance majors (*M* = 20, *SD* = 1.3) | neuroticism | Neuroticism was strongly associated with MPA. | |
| Sarıkaya and Kurtaslan (2018) | Prediction of musical performance anxiety according to music teacher candidates’ perfectionism and self-efficacy beliefs | *N* = 541 undergraduate music teacher candidates | perfectionism | Perfectionism was identified as a substantial predictor of MPA. | |
| Sinden, L. M. (1999) | Music performance anxiety: Contributions of perfectionism, coping style, self-efficacy, and self-esteem | *N* = 138 college students aged between 18 and 50 years (*M* = 21.54, *SD* = 4.99) | perfectionism | Perfectionism was linked to MPA, with the most predictive variables identified as a combination of Concern over Mistakes, Doubts about Actions, Personal Standards and Parental Criticism. | |
| Smith and Rickard (2004) | Prediction of music performance anxiety via personality and trait anxiety in young musicians | *N* = 64 music high school students (*M* = 13.48, *SD* = 1.27) | neuroticism | Neuroticism emerged as a unique significant predictor of MPA. | |
| Spahn et al. (2024) | Personality traits in musicians with different types of music performance anxiety | *N* = 393 musicians (*n =* 89 professional orchestra music (*M* = 43.4, *SD* = 13.2)  , *n* = 195 non-professional orchestra musicians (*M* = 25.2, *SD* = 9.6), *n* = 109 amateur choir singers (*M* = 28.7, *SD* = 13.4)) | neuroticism, extraversion, openness, agreeableness and consciousness | Professional orchestra musicians demonstrated greater openness and conscientiousness than non-professional musicians and amateur choir singers. Musicians with low MPA symptoms, effective coping, and strong self-efficacy generally exhibited lower levels of neuroticism. | |
| Steptoe and Fidler (1987) | Stage fright in orchestral musicians: A study of cognitive and behavioural strategies in performance anxiety | *N* = 146 comprising orchestral players (*n* = 65; *M* = 37, *SD* = 10.5), music students (*n* = 41; *M* = 20.8, *SD* = 2.2), and members of an amateur orchestra (*n* = 40; *M* = 28.9, *SD* = 14.9) | neuroticism | Performance anxiety was positively associated with neuroticism in all age groups. | |
| Stoeber and Eismann (2007) | Perfectionism in young musicians: Relations with motivation, effort, achievement, and distress | *N* = 146 high school musicians aged between 13 and 20 years (*M* = 16.2, *SD* = 1.8) | perfectionism | Negative reaction to imperfection was associated with performance anxiety, whereas striving for perfection was linked to musical effort and achievement. | |
| Thomas and Nettelbeck (2014) | Performance anxiety in adolescent musicians | *N* = 90 secondary school music students aged between 12 and 18 years (*M* = 15.1, *SD* = 1.25) | neuroticism and extraversion | MPA displayed a positive correlation with neuroticism, and conversely, a negative correlation with extraversion. Trait anxiety significantly accounted for the correlations between MPA and both personality traits. | |
